# Transitioning Formamide Solvothermal Syntheses of MOFs to Less Toxic Solvents

**DOI:** 10.1002/chem.202503154

**Published:** 2025-11-10

**Authors:** Julia C. Donovan, Evelyn C. Peterson, Adam J. Matzger

**Affiliations:** ^1^ Department of Chemistry Institution University of Michigan 930 North University Avenue Ann Arbor Michigan 48109‐1055 USA; ^2^ Macromolecular Science and Engineering Program Institution University of Michigan 930 North University Avenue Ann Arbor Michigan 48109‐1055 USA

**Keywords:** crystal growth, solvent effects

## Abstract

Metal‐organic frameworks (MOFs) are most commonly synthesized in toxic formamide solvents. Though some progress has been made for certain classes, zinc carboxylate MOFs are particularly reliant on formamide solvents. Transitioning away from these solvents is difficult because they serve multifaceted roles in MOF formation chemistry and phase direction. Herein, the synthetic factors required for the effective removal of formamide solvents across various classes of MOFs are investigated. Complete formamide removal is achieved for IRMOF‐5, IRMOF‐3, HKUST‐1, and Zn‐MOF‐74. The function of formamide solvents in MOF synthesis is replicated using a system comprising tetrahydrofuran (THF) and a base additive. When designing MOF syntheses without formamide solvents, the solubility of both the organic linker and metal salt, along with the use of a base possessing sufficient p*K*a to deprotonate multitopic linkers, emerge as critical determinants of successful MOF formation. These insights provide a foundation for designing new sustainable MOF syntheses.

## Graphical abstract

Metal‐organic Frameworks (MOFs) are commonly synthesized in toxic formamide solvents. This study investigates the synthetic factors required for effective removal of formamides across various classes of MOFs, providing a foundation for designing new sustainable MOF syntheses. Metal and linker solubility, as well as presence of base capable of deprotonating the linker, emerge as critical determinants of MOF phase formation success.

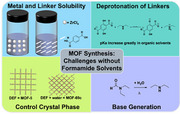

## 1 Introduction

Metal–organic frameworks (MOFs) are widely regarded as critical materials for advancing technologies central to more sustainable energy usage^[^ ^1^ ^]^ and environmental remediation.^[^ ^2^, ^3^ ^]^ However, their syntheses remain largely dependent on costly and toxic formamide‐based solvents.^[^ ^4^ ^]^ This reliance not only raises significant environmental and economic concerns but also limits the applicability of MOFs in areas such as water purification and separations where residual solvent contamination poses serious risks^[^ ^5^ ^]^ and constrains their broader adoption in clean energy systems, efficient separations, and healthcare technologies.

The restrictions on dimethylformamide (DMF)^[^ ^6^ ^]^ in Europe underscores the need to eliminate formamide solvents from MOF synthesis. Yet progress has been limited, primarily due to an incomplete mechanistic understanding of how formamides consistently facilitate the formation of so many desirable MOF phases. The prevailing hypothesis is that formamides serve a dual role: they effectively solubilize organic linkers and metal salts,^[^ ^7^ ^]^ many of which are insoluble in common solvents, and formamides undergo hydrolysis to gradually release dialkylamine base and formic acid.^[^ ^8^ ^]^ The base generated in situ deprotonates the linker, enabling coordination with metal ions and enabling framework assembly, while a highly polar solvent promotes reversibility that is essential for growing crystals with minimal defects. Though it is well established that solvent choice affects properties such as surface area and crystal form,^[^ ^9^, ^10^ ^]^ it remains unclear whether formamides act as structural templates, similar to the templating role in zeolite synthesis,^[^ ^11^ ^]^ or if MOF crystallization can be achieved using common organic solvents, such as ethanol or tetrahydrofuran (THF), provided that linker solubility and deprotonation conditions are met.

Although some MOFs have been successfully synthesized in non‐formamide solvents, these are typically restricted to a narrow range of structurally similar frameworks, and mechanistic insights into why particular solvents succeed remain limited; this hinders proliferation of non‐formamide MOF syntheses, especially for zinc carboxylate MOFs. For instance, N,N‐diethyltoluamide is effective for synthesizing zinc carboxylate MOFs but does not facilitate the formation MOF‐74 or UiO‐type frameworks.^[^ ^12^ ^]^ Sulfolane supports the synthesis of UiO‐66, ZIF‐8, and HKUST‐1.^[^ ^13^ ^]^ MOF‐74 can be synthesized using a solution of THF and sodium hydroxide,^[^ ^14^ ^]^ but high concentration addition of strong base compromises reversibility and results in overall poor crystal quality.^[^ ^15^ ^]^ HKUST‐1 and Zn‐MOF‐74 can be prepared in a methanol/dimethyl sulfoxide (DMSO) mixture.^[^ ^16^ ^]^ HKUST‐1 has been reported to crystallize in ethanol^[^ ^17^ ^]^ as well as in ethanol–water mixtures. ^[^ ^18^ ^]^ Similarly, ZIF‐8 can be synthesized in pure methanol or methanol–water mixtures.^[^ ^19^ ^]^ Beyond organic solvents, several MOFs have been synthesized in aqueous media. Aqueous syntheses of MOF‐74 have been documented in multiple studies^[^ ^20^, ^21^, ^22^ ^]^ with certain reports achieving materials exhibiting high surface areas.^[^ ^23^ ^]^ UiO‐66 and its derivatives have also been prepared in water; however, these syntheses often result in poor crystallinity and, in some instances, with impurity phases.^[^ ^24^ ^]^

**Figure 1:**
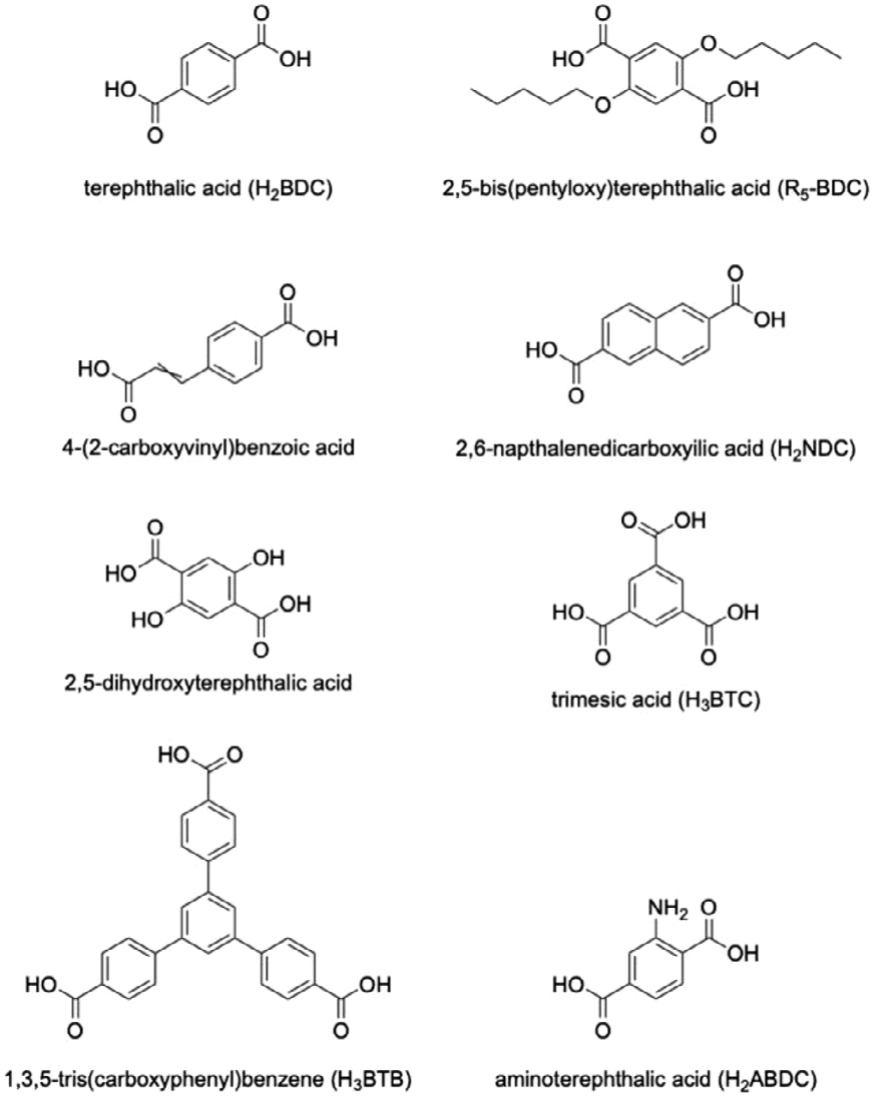
Organic linkers used in this study for MOF synthesis.

While the mechanism of MOF formation often invokes formamide hydrolysis, each MOF class presents distinct synthetic challenges when transitioning to non‐formamide solvents. Moreover, linkers vary in solubility and acidity, which can significantly influence the success of MOF assembly. The MOF‐74 series features phenols that are difficult to deprotonate outside highly polar environments, complicating their synthesis in non‐formamide solvents. Shifting away from formamide‐based systems can be daunting as published protocols frequently report only successful conditions, often without mechanistic rationale or discussion of failed attempts. This study adopts a mechanistic approach to explore key considerations for developing MOF syntheses (the linkers explored in this study are shown in Figure 1) in alternative solvent systems. Beyond practical benefits of reducing reliance on toxic solvents, this work provides insight into the fundamental role of solvent environments in MOF formation, potentially facilitating more scalable syntheses of diverse MOF frameworks.

## 2 Results and Discussion

Zinc carboxylate MOFs, such as MOF‐5, are notably sensitive to synthetic conditions. Despite the successful synthesis of various classes of MOFs in aqueous media, zinc carboxylate MOFs have not been reported. This absence is likely attributable to their pronounced susceptibility to phase conversion, even in the presence of low concentrations of water in formamide‐based systems, rendering aqueous routes largely unviable. Their formation is largely limited to formamide solvents with only a few documented syntheses reported in alternative solvents,^[^ ^12^, ^25^ ^]^ most of which still rely on structurally related amide‐based solvents. Although syntheses in pure alcohol have been demonstrated for other frameworks, such as ethanol‐based synthesis for HKUST‐1 and methanol‐based synthesis for ZIF‐8, analogous approaches employing pure ethanol or methanol have not been reported for zinc carboxylate MOFs. Beyond the choice of solvent, parameters such as water content and pH are equally critical. Both insufficient and excessive water,^[^ ^26^ ^]^ as well as reaction conditions that are too acidic^[^ ^27^ ^]^ or too basic, typically fail to yield the target zinc carboxylate framework. Common additives and modulators used successfully in other MOF systems, such as formic acid^[^ ^28^ ^]^ or hydrochloric acid, tend to inhibit the formation of zinc carboxylate MOFs. Furthermore, the choice of counterion in the metal salt has been shown to influence phase.^[^ ^27^, ^28^ ^]^ Even minor deviations from optimized protocols can divert the reaction toward alternative phases.^[^ ^29^ ^]^ This sensitivity presents a particular challenge for developing routes that do not rely on formamide solvents, especially when so few other variables can be adjusted. An added complication lies in the formation of the secondary building unit (SBU) of the framework, which features a μ_4_‐oxo, generally derived from water.^[^ ^30^ ^]^ Determining a viable solvent alternative to formamides proved challenging. A mixture containing a common ratio of zinc nitrate hexahydrate (Zn(NO_3_)_2_·6H_2_O) and terephthalic acid (H_2_BDC) with the simple substitution of diethylformamide (DEF) for ethyl acetate, THF, acetonitrile, or ethanol led to no MOF‐5 formation (see Supporting Information). However, the absence of MOF‐5 is expected as no base was present to facilitate linker deprotonation. It was hypothesized that by pairing a base additive with a non‐formamide solvent, it may be possible to replicate the functional role traditionally played by formamides. Adding 1 equiv. of diethylamine relative to linker, a combination that yields MOF‐5 in DEF,^[^ ^31^ ^]^ resulted in no MOF‐5 formation with ethyl acetate, THF, acetonitrile, or ethanol.

Faced with the failure of single solvents, the investigation turned to systematically screening cosolvents in combination with DEF, incrementally increasing the cosolvent concentrations until MOF‐5 phase formation ceased. Studies focusing on minimizing the volume of formamides through a cosolvent system are sparse with one study by Biemmi et al.^[^ ^27^ ^]^ examining DEF‐based solvent mixtures with mesitylene, toluene, and chlorobenzene. They found that among these, only mesitylene facilitated MOF‐5 phase formation when Zn(NO_3_)_2_·6H_2_O was used but in the presence of a majority DEF relative to mesitylene. Though the rationale behind using the aromatic cosolvents originated from a BASF patent,^[^ ^32^ ^]^ from solubility considerations of the reactants it is highly unlikely that a pure aromatic solvent would ever be able to produce most MOFs. Besides cosolvent systems, formamide minimization has been accomplished in select cases by using high‐concentration reaction conditions to form the CORN‐1 and CORN‐6 MOF series^[^ ^33^ ^]^ as well as hafnium and zirconium MOFs, including the UiO series.^[^ ^34^ ^]^ Seeded high concentration syntheses have also been utilized to form Mg‐MOF‐74, DMOF‐1, and UiO‐66.^[^ ^35^ ^]^ While these approaches effectively reduce the overall amount of formamide used, it does not allow for complete elimination of formamide solvents. These gaps motivated a broader screening of cosolvents to identify potential replacements for formamides and determine which solvents are compatible with MOF synthesis.

**Figure 2:**
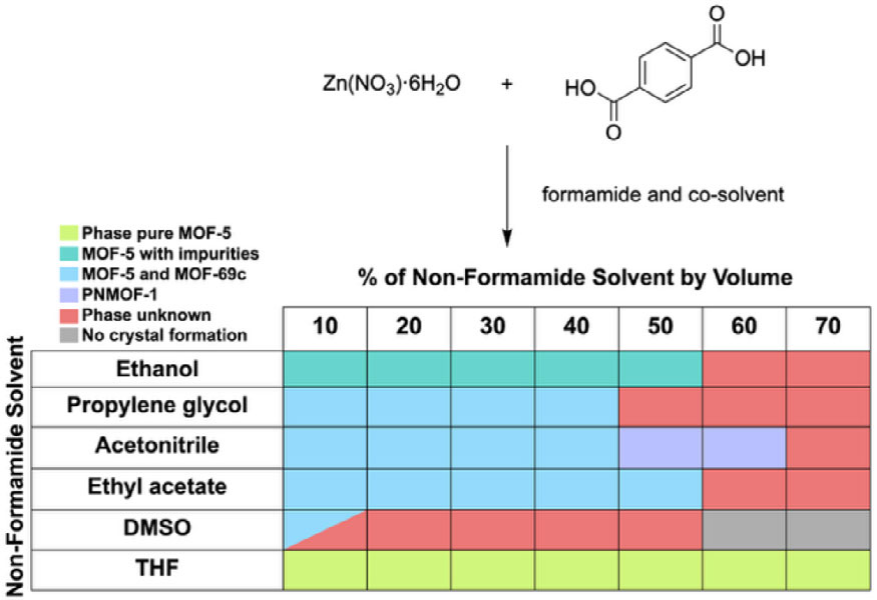
Co‐solvent systems of various organic solvents tested, and the crystal phase that was detected in each synthesis by PXRD with the general reaction scheme shown above.

MOF‐5 is known to undergo conversion to MOF‐69c under humid conditions.^[^ ^36^ ^]^ When additional water is introduced into a precursor solution containing Zn(NO_3_)_2_·6H_2_O and H_2_BDC in DEF, MOF‐69c is the major product. It is understood that both phases share a common formation pathway:^[^ ^37^ ^]^ solvent hydrolysis driving linker deprotonation, with the presence of excess water diverting the phase to MOF‐69c. Because water content must be carefully controlled in a MOF‐5 synthesis, all organic solvents employed here were dry as verified by Karl Fischer titration of < 1,000 µg/mL. Ethanol, commonly used in the synthesis of MOFs, such as HKUST‐1 and MOF‐74, was one of the first solvents investigated. At concentrations of 10% ethanol by volume (relative to DEF) to 50% ethanol, powder X‐ray diffraction (PXRD) patterns revealed the appearance of MOF‐5 peaks. Light microscopy of product from the 10% ethanol solvent system revealed the formation of clear cubic crystals consistent with MOF‐5 as well as opaque cubic, plate‐like, and needle‐like crystals (see Supporting Information). The formation of these opaque crystals was consistently observed up to 50% ethanol. Attempts to solve the structure of these unidentified phases via single‐crystal X‐ray diffraction were unsuccessful. These nontransparent phases are hypothesized to result not from salt formation, but rather from MOF phase collapse, as PXRD data across all ethanol concentrations lacked significant high‐angle peaks characteristic of salt phases. Furthermore, the transition of zinc carboxylate crystals from transparent to opaque is commonly indicative of framework collapse. The consistent presence of impurities and poor crystal quality suggests that, despite the established role of ethanol in other MOF systems,^[^ ^38^ ^]^ it is ineffective at promoting pure zinc carboxylate MOF phase formation even at low concentrations (Figure 2). Propylene glycol, another alcohol‐based solvent, was also evaluated for its potential to promote MOF phase formation. PXRD identified the resulting phase as a mixture of MOF‐5 and MOF‐69c at 10% propylene glycol. MOF‐5 peaks were observed in mixtures containing up to 40% propylene glycol. At a concentration of 50%, a phase unknown formed by PXRD. Similar to the ethanol syntheses, light microscopy revealed a mixture of crystal morphologies, including cubic, plate, and needle‐shaped, comprising both transparent and opaque colorless crystals. It should be noted that the PXRD patterns at 50% concentration exhibited poor reproducibility between synthetic trials, making it difficult to definitively assign the phase mixture. Beyond concentrations of 50%, single crystals ceased to form, and a crystalline powder was obtained instead. Given these inconsistencies, and the inability of propylene glycol to selectively promote MOF‐5 formation, further structural characterization was deprioritized in favor of identifying more effective solvent systems. These findings suggest that polar protic solvents may inhibit the selective formation of zinc carboxylate MOFs, possibly through solvolysis of the DEF, which would rapidly generate base and account for the poor crystal quality observed in these cosolvent systems. Accordingly, a series of polar aprotic solvents was subsequently explored to better support phase‐pure MOF‐5 formation.

Acetonitrile, which has a dielectric constant (36.6 at 20 °C)^[^ ^39^ ^]^ similar to that of DEF (29.6 at 20 °C),^[^ ^40^ ^]^ showed consistent results in the majority of trials. At concentrations of 10%–40% in DEF, a mixture of MOF‐69c and MOF‐5 was obtained, whereas concentrations between 50%–60% yielded PNMOF‐1 with impurities. This latter MOF is known to emerge from MOF‐5 reagents under low‐water conditions (<10,000 ppm),^[^ ^41^ ^]^ yielding a framework characterized by a distorted zinc hourglass cluster rather than a μ_4_‐oxo, supporting the notion that a sufficient concentration of water is necessary for μ_4_‐oxo formation. In the acetonitrile experiments, the absence of the μ_4_‐oxo cannot be attributed to a lack of water, as the metal precursor used, (Zn(NO_3_)_2_·6H_2_O), itself highly hydrated, contributes > 10,000 ppm. Instead, the formation of PNMOF‐1 under increasing proportions of acetonitrile at the expense of the formamide solvent suggests suppression of the water deprotonation pathway required for μ_4_‐oxo formation. The inhibition of μ_4_‐oxo generation under these conditions highlights the challenges of identifying solvent systems capable of supporting the Zn_4_O(CO_2_R)_6_ cluster formation and underscores that polarity matching alone is insufficient to predict successful MOF formation. Overall, while acetonitrile displays partial compatibility with zinc carboxylate systems (no formation of opaque crystals was observed), it offers poor phase selectivity. Ethyl acetate was also evaluated as a cosolvent and demonstrated moderate reproducibility, though the overall performance was suboptimal. Mixtures of MOF‐69c and MOF‐5 were observed across a range of 10%–50% ethyl acetate by volume with a distinct, unidentified phase emerging at 60%–70%. This phase differs from previously encountered unknown phases both with other solvent systems and the unknown phase that appeared when using a 100% ethyl acetate system. Attempts to obtain crystallographic data from this new phase were unsuccessful. DMSO was also selected for investigation as it offers complete solubility of the H_2_BDC linker and has been shown to promote MOF phase formation in some systems.^[^ ^42^, ^43^ ^]^ However, it proved ineffective for zinc carboxylate MOFs. Varying the amount of DMSO in attempts to synthesize MOF‐5 consistently failed to yield phase pure MOF, even at low concentrations (10% DMSO). Instead, a mixture of MOF‐5, MOF‐69c, and a third phase formed, by PXRD (see Supporting Information). Though poorly diffracting, a preliminary structure for the third phase reveals a zinc hourglass SBU that it is distinct from PNMOF‐1 in the space group P 2_1_/c with lattice constants *a * =  13.520 Å, *b * =  9.755 Å, *c * =  18.143 Å, *α* = ***γ*** = 90, and *β* = 110.379. Increasing the concentration of DMSO beyond 10% inhibited the formation of MOF‐5, resulting instead in the appearance of unidentified crystalline phases. At DMSO concentrations above 20%, the crystallization kinetics were significantly slowed with crystal formation requiring 36 to 48 hours. At even higher DMSO content (60%–70%), no crystalline products were observed within 48 hours. Compared to formamide solvents, DMSO has a significantly higher polarity and stronger coordinating ability, which may disrupt the metal–linker interactions required for MOF‐5 assembly.

Among the cosolvents tested, THF emerged as the most promising. Phase‐pure MOF‐5 was obtained at THF concentrations as high as 70%. This is particularly noteworthy given that THF not only supports MOF‐5 formation but also effectively dissolves a range of metal salts and organic linkers, including 2,4‐dihydroxyterephthalic acid (H_4_DOBDC), trimesic acid (H_3_BTC), and 1,3,5‐tris(4‐carboxyphenyl)benzene (H_3_BTB). These properties position THF as a highly versatile solvent for replacement of the formamide solvent across different classes of MOFs. Although its use in the synthesis of MOF‐74 has been reported only in a limited number of cases,^[^ ^14^, ^15^, ^44^ ^]^ these instances suggest that THF imparts sufficient polarity to the MOF solvent system to facilitate reversible protonation and deprotonation of the linker. Further investigation into the coordination behavior of THF with Zn^2^⁺, as well as its influence on linker deprotonation, informs efforts to identify alternative solvents capable of supporting similar reactivity. When comparing THF to formamide‐based solvents, the Pfizer solvent selection guide classifies DMF as *undesirable* and THF as *usable*.^[^ ^45^ ^]^ Toxicological studies further support this distinction: THF has been shown to exhibit low overall toxicity, with no evidence of developmental or reproductive toxicity in rat models.^[^ ^46^ ^]^ In contrast, DMF has been demonstrated to induce both developmental and reproductive toxicity in rats.^[^ ^47^ ^]^

**Figure 3:**
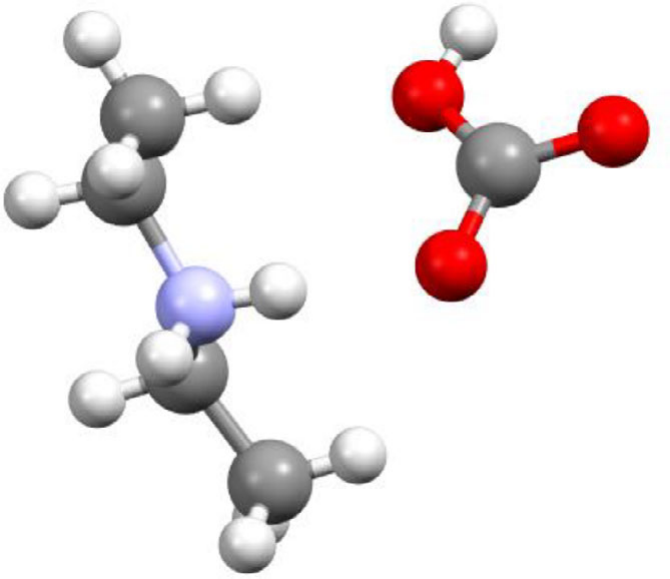
Crystal structure of diethylammonium bicarbonate.

Having identified a promising MOF‐synthesis solvent to replace formamides, attention turned to selecting an appropriate base source to allow complete removal of formamides. Solvothermal MOF syntheses are preferred for producing high‐surface‐area materials, largely due to the slow release of base that promotes controlled‐ crystal growth.^[^ ^48^ ^]^ In contrast, base addition syntheses typically result in immediate precipitation of the powder form rather than single crystals, significantly compromising material quality, including surface area and, by extension, the capacity of the MOF for gas storage.^[^ ^49^ ^]^ Although diethylamine is a viable base, an alternative source was sought that would slow down crystal formation. Diethylammonium bicarbonate (DEAB) was selected as the base source (Figure 3). DEAB is thermally unstable and decomposes upon heating into diethylamine, carbon dioxide, and water. Carbon dioxide is known to have negligible influence on MOF synthesis,^[^ ^50^ ^]^ whereas diethylamine and water are both known to accelerate MOF formation. More importantly, DEAB avoids the introduction of acidic components that could interfere with zinc carboxylate MOFs and eliminates formate anions (one of the products of formamide hydrolysis), which are known to influence phase formation,^[^ ^28^ ^]^ slow down formation kinetics, and introduce defects into MOFs.^[^ ^51^, ^52^ ^]^ Thus, DEAB emerged as a base source that could promote crystallinity in non‐formamide solvent systems.

Attempts to synthesize MOF‐5 in pure THF using H_2_BDC posed a significant challenge due to the poor solubility of the linker. H_2_BDC exhibits limited solubility in most common organic solvents, even at elevated temperatures. While some MOFs, such as MIL‐101, are known to form under heterogeneous conditions,^[^ ^53^ ^]^ attempts to synthesize MOF‐5 under constant time, temperature, and zinc:linker stoichiometry from a reported literature synthesis,^[^ ^54^ ^]^ varying only the solvent to 100% THF and introducing DEAB as the base source, failed. The THF used in these experiments contained < 1,000 µg/mL of water by Karl Fischer titration, which is approximately the amount of residual water in DEF after being dried overnight on 4 Å sieves, and thus, no additional drying was conducted. The resulting phase was identified as isostructural to [NH_2_Et_2_]_2_[Zn_3_(BDC)_4_]·2.5 DEF (CSD refcode: GATBUZ),^[^ ^55^ ^]^ which does not contain a μ_4_‐oxo unit. Although DEAB was able to decompose to generate base and deprotonate the linker, Zn(NO_3_)_2_·6H_2_O and H_2_BDC combined in a typical ratio did not yield the MOF‐5 phase. It was hypothesized that this alternative phase arose either from poor solubility of the linker or from the inability of THF to facilitate μ_4_‐oxo formation. To determine if linker solubility was the barrier preventing MOF‐5 formation, 2,5‐bis(pentyloxy)terephthalic acid (R_5_‐BDC), a derivative with alkoxy chains that imparts solubility in organic solvents, was employed and has been previously used to synthesize IRMOF‐5.^[^ ^56^ ^]^ Substituting DEF with THF and using DEAB as the base source led to the formation of IRMOF‐5 as a crystalline powder, which represents one of the few examples of a zinc carboxylate MOF synthesized without formamides. Diethylamine, when used as the base source, also yielded IRMOF‐5; however, the resulting material consistently exhibited smaller crystal sizes by light microscopy (see Supporting Information). The larger crystals in the DEAB synthesis are attributed to its more gradual base generation. The synthesis of IRMOF‐3 was attempted using a similar approach to IRMOF‐5 to explore the importance of linker solubility. The linker of IRMOF‐3, 2‐aminoterephthalic acid (H_2_ABDC), is soluble in THF, and while desired phase formation was achieved, the resulting material was impure as evidenced by PXRD as a second phase was observed (see Supporting Information). Attempts to mitigate the unwanted phase by optimization of synthetic factors proved unsuccessful (see Supporting Information). These results reinforce the broader conclusion that MOF formation is not a universally transferable process but one that often requires system‐specific optimization even among structurally similar MOFs.

To evaluate whether MOFs synthesized using THF and DEAB exhibit porosity, Brunauer–Emmett–Teller (BET) surface area analysis was performed. Because experimental surface area data for IRMOF‐5 has not been reported in the literature, attention was directed toward IRMOF‐3 for surface area evaluation. Multiple attempts to solvent exchange and activate IRMOF‐3 consistently yielded low surface areas, approximately 270 m^2^/g when synthesized using THF as the solvent and DEAB as the base source. The measured mass for BET contained the secondary phase impurity that is observable via PXRD, which may contribute to an artificially inflated sample mass, thereby lowering the calculated surface area per gram if the secondary phase is nonporous. Furthermore, the BET isotherm revealed the presence of some mesoporous and macroporous material, which could correspond to defective IRMOF‐3 or to the secondary phase. To differentiate between these possibilities, crystals were synthesized in THF with DEAB. Upon exposing the MOF mixture to a saturated solution of Nile Red, followed by washing, cubic crystals adopted a blue coloration (IRMOF‐3) while the other phase remained an unchanged brown (see Supporting Information), indicating that the secondary phase is nonporous. The uptake of IRMOF‐3 crystals indicates successful dye encapsulation and confirms that, the mesoporous and macroporous material observed by BET likely corresponds to internal defects of IRMOF‐3, as has been observed with postsynthetic modifications;^[^ ^57^ ^]^ however, the THF‐DEAB synthetic method is clearly capable of yielding porous MOFs.

**Figure 4:**
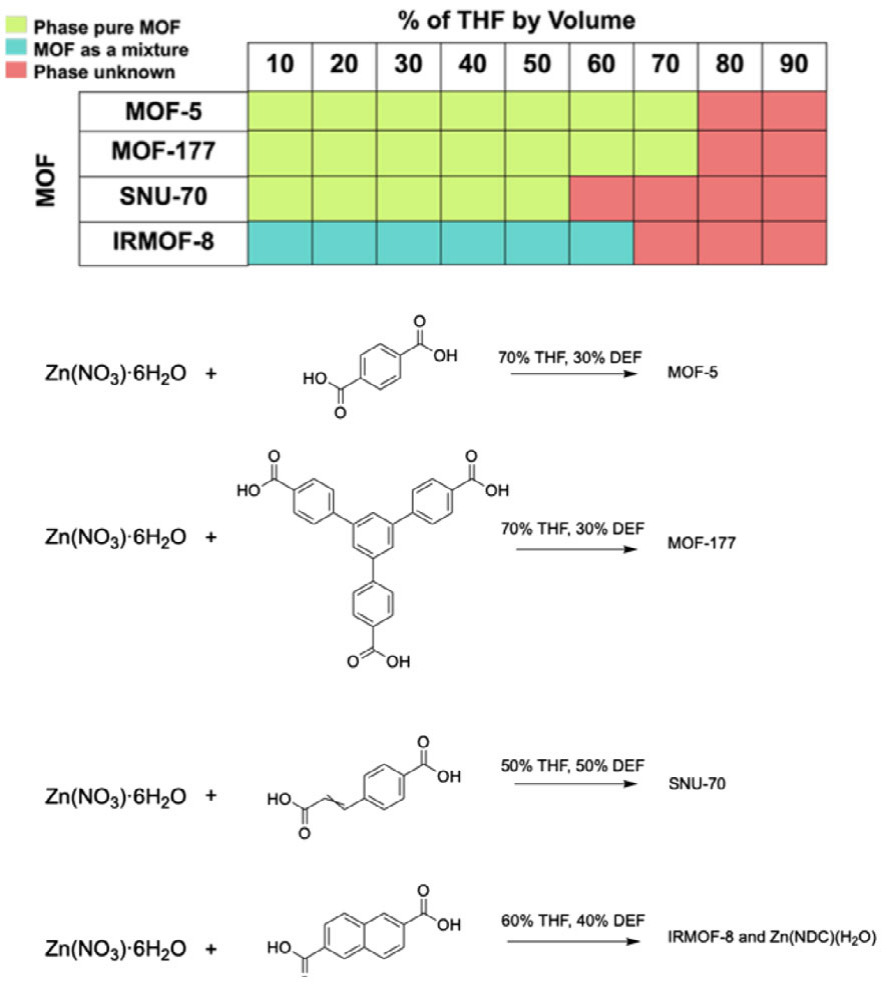
THF and DEF cosolvent systems of various zinc carboxylate syntheses, and the phases detected by PXRD. Below are the reaction schemes for the MOF syntheses.

Linker solubility is not the sole factor governing phase formation in THF. MOF‐177, which is constructed from deprotonated H_3_BTB, could not be synthesized in 100% THF despite the solubility of its linker. Attempts to synthesize MOF‐177 using different zinc salts failed under a variety of conditions, including changes in temperature, reaction time, and metal‐to‐linker ratios. Given that IRMOF‐5 and IRMOF‐3 were successfully synthesized in THF, it is unlikely that the failure of MOF‐177 to form is due to limitations in μ_4_‐oxo formation. One explanation may lie in the structure of H_3_BTB, a tritopic linker with an extended π‐conjugated system. Upon deprotonation in THF, H_3_BTB likely acquires a high negative charge, which may trigger premature precipitation before MOF assembly. Using THF as a cosolvent with DEF enables formation of MOF‐177 at THF concentrations as high as 70% by volume (Figure 4). This result demonstrates that while THF can effectively reduce reliance on formamide solvents, it does not offer a complete replacement. Ensuring that fully deprotonated H_3_BTB remains soluble via an optimized cosolvent system may be crucial for producing MOF‐177 under non‐formamide conditions. Other zinc carboxylate MOFs, such as IRMOF‐8 and SNU‐70, involve linkers that are insoluble in THF, precluding their synthesis in pure THF.^[^ ^20^ ^]^ Cosolvent systems of THF and DEF were employed to determine the amount of formamide solvent that could be decreased. For SNU‐70, crystalline MOF was yielded up to 50% THF by volume with higher concentrations resulting in no crystalline product. In the case of IRMOF‐8, the MOF was observed up to and at 60% THF along with an additional phase. The other phase was identified as Zn(NDC)(H_2_O),^[^ ^58^ ^]^ which formed exclusively beyond 60% THF concentrations. Overall, these findings demonstrate the potential of THF to significantly reduce the amount of formamide utilized with complete removal of the formamide solvent in the cases of IRMOF‐3 and IRMOF‐5.

**Figure 5:**
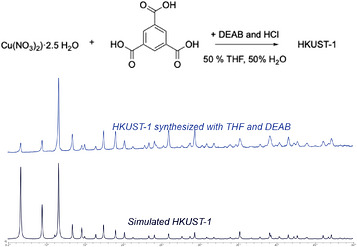
The synthesis of HKUST‐1 using THF and DEAB and HCl (blue) compared to simulated HKUST‐1 (black).

Extending the use of the THF‐DEAB system to MOFs besides zinc carboxylates, the synthesis of UiO‐66‐NH_2_ was attempted with no crystalline material observed in any syntheses (see Supporting Information). Unlike the challenges observed in zinc carboxylate MOFs where linker solubility is often the limiting factor, the primary obstacle in synthesizing UiO‐66‐NH_2_ is believed to lie in the poor solubility of the zirconium(IV) salts in THF. Applying the THF‐DEAB system in the synthesis of HKUST‐1 yielded promising results. The organic linker H_3_BTC is readily soluble in THF. HKUST‐1 is typically synthesized in a mixed solvent system comprising water, ethanol, and DMF.^[^ ^59^ ^]^ Replacing the DMF and ethanol with THF and DEAB lead to the formation of HKUST‐1. Systematic variation of water content in the THF‐based synthesis revealed that HKUST‐1 formed when water comprised 10%–50% of the solvent mixture by volume. No MOF was obtained in the absence of water or when water exceeded 50%; both conditions lead to the formation of unidentified phases. The importance of water in both the DMF and THF systems is evident where it likely aids in achieving solubility of the highly charged states of the tritopic linker. To further modulate crystallization kinetics, HCl was introduced to the THF‐DEAB system. At 85 °C, HCl inhibited MOF formation, but increasing the temperature to 100 °C enabled the successful synthesis of HKUST‐1 (Figure 5).

HKUST‐1 is notable for its coordinatively unsaturated sites, which bind strongly to solvents and require harsh activation conditions for complete solvent removal.^[^ ^60^ ^]^ Given that THF has a similar surface tension to dichloromethane,^[^ ^61^, ^62^ ^]^ a solvent from which activation is often feasible, it was hypothesized that full solvent exchange might not be necessary. When HKUST‐1 was synthesized in a 50:50 THF:water mixture with DEAB and HCl, washed three times with THF, and activated under vacuum at 150 °C, the material displayed the expected color change from light blue to purple upon activation, indicating successful removal of coordinated solvent.^[^ ^63^ ^]^ Although this color change occurred within a few hours, vacuum was maintained overnight to align with standard activation procedures. The resulting BET surface area is 1250 m^2^/g, which, while lower than the best literature values,^[^ ^64^ ^]^ still reflects a high level of porosity and structural integrity, demonstrating the viability of this synthesis route. The HKUST‐1 synthesis in THF is a more robust synthesis than that of IRMOF‐5 as variations in water content and the use of HCl still afforded the same phase, potentially offering a more flexible platform for surface area optimization.

**Figure 6:**
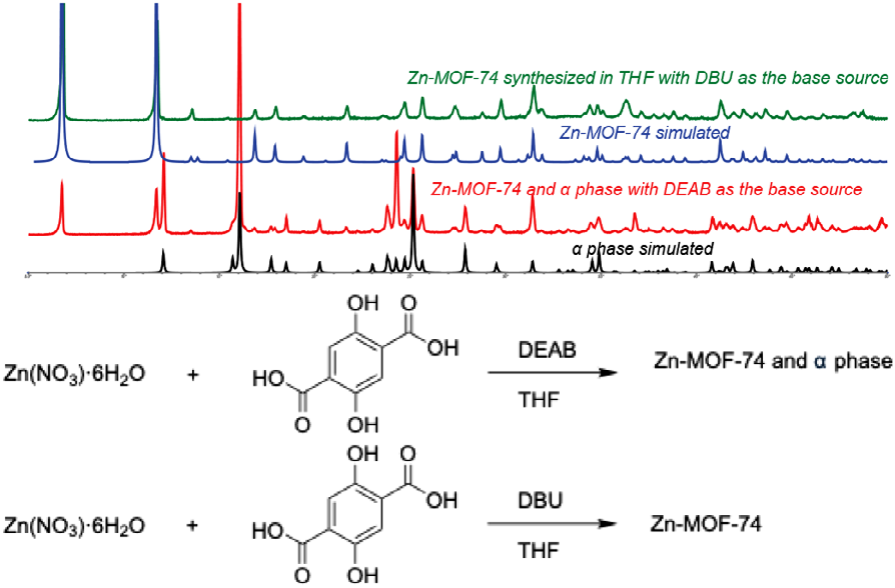
Synthesis of Zn(NO_3_)_2_ ·6H_2_O and H_4_DOBDC in THF with 3 equiv. DEAB as the base source (red) resulting in a mixture Zn‐MOF‐74 and the α phase compared to the simulate α phase (green) and synthesis of Zn(NO_3_)_2_ ·6H_2_O and H_4_DOBDC in THF with 3 equiv. DBU as the base source (blue) resulting in Zn‐MOF‐74 compared to simulated Zn‐MOF‐74 (black). The synthesis scheme depicting the syntheses are shown below the PXRD patterns.

Finally, to evaluate the broader applicability of the THF‐DEAB system, it was investigated in the synthesis of Zn‐MOF‐74. Previous studies have demonstrated that Zn‐MOF‐74 can be produced with high space–time yields,^[^ ^21^ ^]^ positioning it as an important model for exploring routes toward more industrially feasible MOF syntheses. Similar to the synthesis of HKUST‐1, water content was determined to be a critical factor in promoting the synthesis of Zn‐MOF‐74. Adjusting the water content to 30% increased the overall polarity of the solvent system and modulated the p*K*a of the linker to enable partial formation of the desired phase (lower or higher water concentrations failed to form any Zn‐MOF‐74, and thus, subsequent trials were conducted in a solvent mixture of 70% THF and 30% water). Under these conditions, PXRD revealed the coexistence of Zn‐MOF‐74 and a secondary phase, referred to in literature as the α phase (Figure 6).^[^ ^65^ ^]^

**Figure 7:**
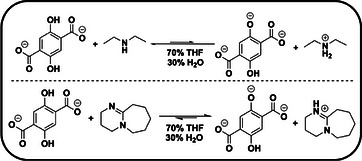
Deprotonation pathway of the phenols in THF depending on the base utilized. When diethylamine is used, the equilibrium lies toward the left. When DBU is used, the equilibrium lies toward the right.

A key challenge in synthesizing the MOF‐74 family in non‐formamide solvents lies in achieving the effective deprotonation of the phenols on the linker, which possess higher p*K*a values than carboxylic acids. The α MOF phase contains a ZnO_4_ cluster with two of the oxygens coordinated through the deprotonated carboxylic acids of the linker and the other two oxygen originating from water. Notably, the phenols remain protonated in this phase. Altering the reaction temperature did not improve phase purity; higher temperatures resulted in the formation of a distinct β phase, which is previously reported but structurally uncharacterized due to the small size of the resulting crystals.^[^ ^65^ ^]^ Lower temperatures yielded the α phase exclusively. Increasing the DEAB concentration failed to yield exclusively MOF‐74 with a mixture of the desired MOF and α phase still obtained. Beyond 3.5 equivalents of base, new, unidentified phases were introduced. Both single crystals and a crystalline powder were obtained under these conditions. The single crystals were identified as α phase by single‐crystal X‐ray diffraction. Neither the α, β, nor MOF‐74 phases were observed when analyzing the powder by PXRD. This highlights the central difficulty of synthesizing MOF‐74 in non‐formamide solvents: the efficient deprotonation of the phenol groups. While p*K*a values for phenols and diethylammonium in THF are unavailable, values in acetonitrile offer useful guidance. The p*K*a of phenol increases significantly from 9.99 in water to 18.4 in DMF and up to 29.2 in acetonitrile.^[^ ^66^ ^]^ Diethylamine, the decomposition product of DEAB, has a conjugate acid with a p*K*a of 18.75 in acetonitrile,^[^ ^67^ ^]^ suggesting it is insufficiently basic to fully deprotonate phenols in the THF environment. To test whether a stronger base could improve MOF‐74 formation, 1,8‐diazabicyclo[5.4.0]undec‐7‐ene (DBU) was employed, which, when protonated, has a pKa of 24.31 in acetonitrile (Figure 7).^[^ ^67^ ^]^ After 24 hours at 100 °C in a solvent mixture of 70% THF and 30% water, the β phase was again the dominant product. However, reducing the temperature suppressed formation of this undesired phase. Between 70 °C and 40 °C, Zn‐MOF‐74 became the major product, with higher temperatures associated with the presence of impurities and lower temperatures yielding purer material.These results demonstrate that stronger bases, such as DBU, can overcome the deprotonation barrier associated with phenol‐functionalized linkers and offer a viable pathway for synthesizing MOF‐74‐type materials in non‐formamide solvents.

## 3 Conclusion

This work establishes THF as an effective replacement for a portion or all formamide solvents in various MOF syntheses. By systematically exploring solvent requirements across different MOF classes, it was demonstrated that, while linker solubility is often a key determinant of successful MOF formation, poorly soluble linkers can yield the desired crystal phase with substantially reduced formamide content. Through cosolvent screening, alternative organic solvents that either promote or hinder MOF crystallization were identified, offering further insight into solvent‐MOF interactions. Ultimately, THF combined with a base additive was identified as the most broadly applicable solvent system for synthesizing diverse classes of MOFs, including IRMOF‐5, HKUST‐1, and Zn‐MOF‐74, without relying on conventional formamides. The low surface tension of THF eliminates multistep solvent exchange, allowing for activation from THF and streamlining post‐synthetic processing. Investigations into the MOF‐74 system reveal that the structural identity of the base is less critical than its capacity to deprotonate the linker, suggesting opportunities to design reagents which gradually introduce base into the system. Beyond improving the sustainability and practicality of MOF synthesis, this work advances a deeper understanding of parameters that govern MOF assembly, emphasizing the need for linker and metal salt solubility and outlining the foundation for further innovations in scalable and more environmentally palatable synthetic methods.

## 4 Experimental Methods

### Materials

Zinc nitrate hexahydrate (Zn(NO_3_)_2_ ·6H_2_O, Alfa Aesar, 99%), copper nitrate hemipentahydrate, (Cu(NO_3_)_2_ ·2.5H_2_O, Fisher Scientific), and zirconium chloride (ZrCl_4_, Strem Chemicals, 99%) were used as received. Benzene‐1,4‐dicarboxylic acid (H_2_BDC, Sigma‐Aldrich, >98%), 1,3,5‐tris(carboxyphenyl)benzene (H_3_BTB, Ambeed, >95%), 4‐carboxycinnamic acid (H_2_CCA, Ambeed, >95%), 2,6‐napthalenedicarboxyilic acid (H_2_NDC, TCI Chemical, >98%), 2‐aminoterephthalic acid (H_2_ABDC, Acros Organic, 99%), trimesic acid (H_3_BTC, TCI America, 98%), and 2,5‐dihydroxyterephthalic acid (H_4_DOBDC, TCI America, 98%) were used as received.

N,N‐diethylformamide (DEF, 1PlusChem, 98%) was purified by storage on activated charcoal for 1 week, followed by removal of impurities via silica gel column and storage over 4 Å molecular sieves. THF was purchased from Honeywell. Dimethylsulfoxide (DMSO), ethyl acetate, acetonitrile, propylene glycol, and dimethylformamide (DMF), and ethanol were purchased from Fisher Scientific. To remove residual moisture, solvents were stored over activated 4 Å molecular sieves, and ethanol was stored over 3 Å molecular sieves.

Diethylamine (Acros Organic, 99%), benzene (Fisher Scientific), 1,8‐diazabicyclo[5.4.0]undec‐7‐ene (DBU, 98% Sigma‐Aldrich), hydrochloric acid (HCl, Fisher Scinetific), potassium carbonate (K_2_CO_3_, Fisher Scientific), potassium hydroxide (KOH, Fisher Scientific), 1‐bromopentane (Sigma–Aldrich, 98%), and dimethyl 2,5‐dihydroxy‐1,4 benzenedicarboxylate (Ambeed, 98%) were used as received.

### Methods

**Powder X‐ray diffraction (PXRD)**. Room‐temperature powder X‐ray diffraction was performed on a Rigaku MiniFlex 600 equipped with a *θ*/2*θ* goniometer. Data were collected in Bragg–Brentano geometry using Ni‐filtered Cu Kα radiation (*K*α1  =  1.54 059 Å, *K*α2  =  1.54 441 Å, *K*α2/ *K*α1  =  0.5) operating at 40 kV, and 15 mA. The incident beam was equipped with a variable and fixed (1.25° divergence) slit and a 5° Soller slit. A 5° Soller slit was on the diffracted beam side. X‐ray detection was accomplished with a silicon‐based linear position sensitive D/tex Ultra2 detector operating in 1D scanning mode. MOF samples, finely ground as needed, were packed into the depression of a zero‐background silicon crystal holder. Data were collected between 3°–50° 2*θ*, using a scan rate of 10.0°/min and a step size of 0.01°. Powder patterns were processed using MDI JADE v9.1 software.

**Single Crystal X‐Ray Diffraction (SCXRD)**. SCXRD data were collected using a Rigaku XtaLAB Synergy‐S X‐ray diffractometer configured in a κ goniometer geometry. The diffractometer is equipped with a low‐temperature device and a PhotonJet‐S microfocus Cu source (*λ* = 1.54 187 Å) operated at 50 kV and 1 mA. X‐ray intensities were measured at 293(2) K with a HyPix‐6000HE detector placed 34.00 mm from the sample. The data were processed with CrysAlisPro v44.12a (Rigaku Oxford Diffraction) and corrected for absorption. The structures were solved in OLEX2^[^ ^68^ ^]^ version 1.5‐ac5‐024 using SHELXT^[^ ^69^ ^]^ and refined using SHELXL.^[^ ^70^ ^]^ All nonhydrogen atoms were refined anisotropically with hydrogen atoms placed at idealized positions. Additional structure details can be found in the Supporting Information and the cif file was deposited in the Cambridge Structural Database.^[^ ^71^, ^72^ ^]^

**Water Content Determination**. Karl Fischer titration experiments were performed using a coulometric Mettler Toledo C20. Approximately 1 mL of sample was introduced to the system via syringe transfer, and a 15 second mix time was performed before measurement.

**Surface Area Measurement**. N_2_ sorption experiments were performed on a NOVA e series 4200 surface area analyzer (Quantachrome Instruments, Boynton Beach, Florida, USA). N_2_ (99.999%) was purchased from Cryogenic Gases. For N_2_ measurements, a glass sample cell was charged with ∼30 mg of sample, activated, and analyzed at 77 K. N_2_ sorption isotherms were collected in the NOVA‐win software.

**NMR Spectroscopy**. Proton nuclear magnetic resonance (^1^H NMR) spectra were recorded on Varian vnmrs 500 and Varian vnmrs 400 spectrometers.

**Organic Syntheses**. The following general procedures were followed.

**Synthesis of 2,5‐bis(pentyloxy)terephthalic acid (R_5_‐BDC)**. R_5_‐BDC was synthesized by the following literature procedure^[^ ^73^ ^]^ with slight modifications. Dimethyl 2,5‐dihydroxy‐1,4 benzenedicarboxylate (600 mg, 2.65 mmol) was dissolved in a round bottom flask with 20 mL of DMF via sonication. K_2_CO_3_ (1.50 g, 10.8 mmol) was added under vigorous stirring. 1‐Bromopentane (0.970 mL, 7.83 mmol) was added dropwise to the solution. The round bottom flask was heated to 85 °C overnight. The next morning, the crude reaction was filtered while hot, and the excess solvent was evaporated under vacuum by rotary evaporation at 60 °C. THF (12.5 mL) and 5% KOH solution (12.5 mL) were added to the crude reaction mixture which was heated to reflux overnight. The next morning, the crude reaction mixture was cooled to room temperature then cooled to 0 °C. R_5_‐BDC was precipitated from solution with 10% HCl. The product was filtered and washed 3 × with distilled water. The linker was dried under vacuum and the identity confirmed via ^1^H NMR spectroscopy. (400 MHz, DMSO‐D6): *δ*, 7.25 (s, 2H), 3.97 (t, 4H), 1.70–1.65 (m, 4H),1.42–1.29 (m, 8H), 0.88 (t, 6H) ppm.

**Diethylammonium bicarbonate (DEAB) Synthesis**. DEAB was synthesized by the following literature procedure with slight modifications.^[^ ^74^ ^]^ Diethylamine (1.00 mL, 9.67 mmol) was added to 15 mL of benzene in a round bottom flask. CO_2_ was bubbled through the solvent for 15 minutes while the solution stirred. After 15 minutes, 5 mL of deionized water was added, and the solution became cloudy. The reaction mixture was stirred for an additional 45 minutes. The solvent was removed under dynamic vacuum (0.05 torr) for 6 hours. The product was isolated as a light brown solid with the structure confirmed by SCXRD for the first synthesis and subsequent syntheses by PXRD. ^1^H (500 MHz, D_2_O): *δ* 3.03 (q, J  =  7.3 Hz, 4H), 1.23 (t, J  =  7.3 Hz 6H). ^13^C (126 MHz, D_2_O): *δ* 160.31, 42.19, 10.47. IR (cm^−1^): 2987.73, 2947.23, 2881.66, 2484.39, 2403.39, 1635.37, 1588.6, 1504.23, 1473.37, 1452.64, 1385.14, 1350.43, 1321.99, 1163.85, 1067.42, 1012.94, 972.45, 866.86, 834.08, 809.01, 706.31, 653.76, 497.07, 413.66. Potassium bicarbonate was spiked in as an authentic standard to confirm synthesis of the bicarbonate via ^13^C NMR.

**MOF Syntheses**. The following general procedures were followed.

**Cosolvent testing of H_2_BDC linker**. H_2_BDC (20.0 mg, 0.120 mmol) and Zn(NO_3_)_2_·6H_2_O (100 mg, 0.336 mmol) were added to a total of 2.0 mL of solvent, containing mixed concentrations (see Supporting Information) of DEF and an organic solvent (ethanol, propylene glycol, acetonitrile, ethyl acetate, DMSO, or THF) in a 4 mL vial before being heated to 100 °C for 24 hours.

**Cosolvent testing of H_3_BTB linker**. H_3_BTB (23.4 mg, 0.140 mmol) and Zn(NO_3_)_2_·6H_2_O (48.6 mg, 0.164 mmol) were added to a total of 2.0 mL of solvent, containing mixed concentrations (see Supporting Information) of DEF and THF in a 4 mL vial before being heated to 85 °C for 48 hours.

**Cosolvent testing of H_2_NDC linker**. H_2_NDC (10.0 mg, 0.0462 mmol) and Zn(NO_3_)_2_·6H_2_O (42.9 mg, 0.144 mmol) were added to a total of 2.0 mL of solvent, containing mixed concentrations (see Supporting Information) of DEF and THF in a 4 mL vial before being heated to 85 °C for 36 hours.

**Cosolvent testing of H_2_CCA linker**. H_2_CAA (6.00 mg, 0.0312 mmol) and Zn(NO_3_)_2_·6H_2_O (12.0 mg, 0.050 mmol) were added to a total of 2.0 mL of solvent, containing mixed concentrations (see Supporting Information) of DEF and THF in a 4 mL vial before being heated to 105 °C for 18 hours.

**IRMOF‐3 synthesis**. H_2_ABDC (9.60 mg, 0.0521 mmol) and Zn(NO_3_)_2_·6H_2_O (41.6 mg, 0.159 mmol) were added to THF (2.0 mL) in a 4 mL vial and dissolved through sonication for ∼10 minutes. The solution was then sparged with N_2_ gas for 30 seconds before DEAB (6.20 mg, 0.0458 mmol) was added to the solution. A cap fitted with an additional Teflon insert with a thickness of 1.87 mm was screwed on then heated to 100 °C for 20 hours to form IRMOF‐3.

**IRMOF‐5 synthesis**. R_5_‐BDC (23.0 mg, 0.0680 mmol) and Zn(NO_3_)_2_·6H_2_O (7.00 mg, 0.0235 mmol) were added to THF (2.0 mL) in a 4 mL vial and dissolved through sonication for ∼10 minutes. DEAB (8.00 mg, 0.0592 mmol) or diethylamine (7.00 µL, 0.0680 mmol) was added to the solution. A cap fitted with an additional Teflon insert with a thickness of 1.87 mm was screwed on then heated to 100 °C for 24 hours to form IRMOF‐5.

**HKUST‐1 synthesis**. H_3_BTC (40.0 mg, 0.190 mmol) and Cu(NO_3_)_2_·2.5H_2_O (80.0 mg, 0.344 mmol) were added to THF (1.0 mL) and deionized water (1.0 mL) in a 4 mL vial and dissolved through sonication for ∼10 minutes. HCl (two drops) then DEAB (22.0 mg, 0.162 mmol) were added to the solution. A cap fitted with an additional Teflon insert with a thickness of 1.87 mm was screwed on then heated to 100 °C for 24 hours to form HKUST‐1.

**Zn‐MOF‐74 synthesis**. H_4_DOBDC (10.0 mg, 0.050 mmol) and Zn(NO_3_)_2_·6H_2_O (40.0 mg, 0.134 mmol) were added to THF (1.40 mL) and deionized water (0.60 mL) in a 4 mL vial and dissolved through sonication for ∼10 minutes. DBU (22.5 µL, 0.150 mmol) was added to the solution then heated to 40 °C for 24 hours to form Zn‐MOF‐74.

**Activation of HKUST‐1**. HKUST‐1 was synthesized using the above procedure scaled up to a 10 mL scale. After synthesis, the crystals were filtered and washed 3 × with THF. Once the solvent exchange was complete, the crystals were evacuated under dynamic vacuum (0.05 torr) overnight at 150 °C.

**Activation of IRMOF‐3**. IRMOF‐3 was synthesized using the above procedure scaled up to a 10 mL scale. After synthesis, the crystals were filtered and washed 3 × with THF then 3 × with hexane. Once the solvent exchange was complete, the crystals were evacuated under dynamic vacuum (0.05 torr) overnight at room temperature.

## Conflict of Interest

The authors declare no conflict of interest.

## Supporting information



Supporting Information

## Data Availability

The data that support the findings of this study are available in the supplementary material of this article.
