# Supplementary material for: Transitioning Formamide Solvothermal Syntheses of MOFs to Less Toxic Solvents
Source: Chemistry. 2025 Nov 10;32(29):e03154. doi: 10.1002/chem.202503154 (PMC13451561; doi:10.1002/chem.202503154)
Supplement: Supplementary file 1 [file CHEM-32-e03154-s001.docx]

Transitioning Formamide Solvothermal Syntheses of MOFs to Less Toxic Solvents

Supporting Information

Julia C. Donovan^a^, Evelyn C. Peterson^a^ and Adam J. Matzger^a, b*^

^a^Department of Chemistry, University of Michigan, Ann Arbor, Michigan 48109-1055, United States.

^b^Macromolecular Science and Engineering, University of Michigan, Ann Arbor, Michigan 48109-1055, United States.

Table of Contents

1. Amounts of Co-solvent Utilized
2. Tested MOF Synthesis Conditions
3. Powder X-ray Patterns (PXRD)
4. Crystal Pictures
5. Characterization Data of DEAB
6. N_2_ Isotherms
1. **Amounts of Co-solvent Utilized**

**Table S1**: amount of DEF and co-organic solvents used in the co-solvent systems.

| Amount of DEF (mL) | Amount of organic solvent (mL) | % of co-solvent |
| --- | --- | --- |
| 1.8 | 0.2 | 10 |
| 1.6 | 0.4 | 20 |
| 1.4 | 0.6 | 30 |
| 1.2 | 0.8 | 40 |
| 1.0 | 1.0 | 50 |
| 0.8 | 1.2 | 60 |
| 0.6 | 1.4 | 70 |
| 0.4 | 1.6 | 80 |
| 0.2 | 1.8 | 90 |

**2. Tested MOF Synthesis Conditions**

For all trials below, the synthesis was run in a 4 mL vial with 2 mL of solvent and 2 mL of headspace. Reported mass values reflect approximate amounts used during synthesis. Values are rounded appropriately based on balance precision and are accurate to within ±1 mg unless otherwise stated.

**Table S2**: tested synthesis conditions for IRMOF-5 using 23 mg of R_5_-BDC and 7 mg of Zn(NO_3_)_2_·6H_2_O.

| Solvent | Time (hours) | Base Amount | Temperature (°C) | Phase |
| --- | --- | --- | --- | --- |
| THF | 0.5 | 8 mg DEAB | 40 | Not IRMOF-5 |
| THF | 5 | 8 mg DEAB | 40 | Not IRMOF-5 |
| THF | 0.5 | 8 mg DEAB | 60 | Not IRMOF-5 |
| THF | 5 | 8 mg DEAB | 60 | Not IRMOF-5 |
| THF | 16 | 8 mg DEAB | 60 | Not IRMOF-5 |
| THF | 5 | 8 mg DEAB | 80 | Not IRMOF-5 |
| THF | 16 | 8 mg DEAB | 80 | Not IRMOF-5 |
| THF | 5 | 8 mg DEAB | 100 | Not IRMOF-5 |
| THF | 16 | 8 mg DEAB | 100 | IRMOF-5 |
| THF | 24 | 4 mg DEAB | 100 | IRMOF-5 |
| THF | 24 | 8 mg DEAB | 100 | IRMOF-5 |
| THF | 24 | 10 mg DEAB | 100 | IRMOF-5 |
| THF | 24 | 18 mg DEAB* | 100 | IRMOF-5 |
| THF | 24 | 7.00 μL diethylamine | 100 | IRMOF-5 |

* Using 18 mg of DEAB often gives IRMOF-5 but less consistently than using 8 mg.

**Table S3**: tested synthesis conditions for IRMOF-3 using 4.8 mg of H_2_ABDC.

| Solvent | Time (hours) | Base Amount | Temperature (°C) | Amount of Metal (mg) | Phase |
| --- | --- | --- | --- | --- | --- |
| THF | 16 | 6.2 mg DEAB | 85 | Zinc nitrate 41.6 mg | Not IRMOF-3 |
| THF | 16 | 6.2 mg DEAB | 120 | Zinc nitrate 41.6 mg | Not IRMOF-3 |
| THF | 16 | 3.1 mg DEAB | 100 | Zinc nitrate 41.6 mg | IRMOF-3 but impure* |
| THF | 16 | 2.6 μL diethylamine | 100 | Zinc nitrate 41.6 mg | IRMOF-3 but impure |
| THF | 16 | 1.5 mg DEAB | 100 | Zinc nitrate 41.6 mg | IRMOF-3 but impure |
| THF | 16 | 6.2 mg DEAB | 100 | Zinc nitrate 41.6 mg | IRMOF-3 but impure |
| THF | 16 | 7.8 mg DEAB | 100 | Zinc nitrate 41.6 mg | No IRMOF-3, only impurity |
| THF | 18 | 6.2 μL diethylamine | 100 | Zinc nitrate 41.6 mg | IRMOF-3 but impure |
| THF | 2 | 1.5 mg DEAB | 100 | Zinc nitrate 41.6 mg | IRMOF-3 but impure |
| THF | 16 | 1.5 mg DEAB | 100 | Zinc nitrate 41.6 mg | IRMOF-3 but impure |
| THF | 20 | 1.5 mg DEAB | 100 | Zinc nitrate 41.6 mg | IRMOF-3 but impure |
| THF | 22 | 1.5 mg DEAB | 100 | Zinc nitrate 41.6 mg | IRMOF-3 but impure |
| THF | 2 | 6.2 μL diethylamine | 100 | Zinc nitrate 41.6 mg | IRMOF-3 but impure |
| THF | 5 | 6.2 μL diethylamine | 100 | Zinc nitrate 41.6 mg | IRMOF-3 but impure |
| THF | 24 | 6 μL diethylamine | 100 | Zinc nitrate 41.6 mg | IRMOF-3 but impure |
| THF | 24 | 1.5 mg DEAB | 100 | Zinc nitrate 7.8 mg | Not IRMOF-3 |
| THF | 24 | 1.5 mg DEAB | 100 | Zinc nitrate 15.6 mg | Not IRMOF-3 |
| THF | 24 | 1.5 mg DEAB | 100 | Zinc nitrate 31 mg | No IRMOF-3, only impurity |
| THF | 24 | 1.5 mg DEAB | 100 | Zinc nitrate 62 mg | No IRMOF-3, only impurity |
| THF | 24 | 1.5 mg DEAB | 100 | Zinc nitrate 124 mg | No IRMOF-3, only impurity |
| THF | 24 | 1.5 mg DEAB | 100 | Zinc triflate 14 mg | Not IRMOF-3 |
| THF | 24 | 1.5 mg DEAB | 100 | Zinc triflate, 7 mg | Not IRMOF-3 |
| THF | 24 | 1.5 mg DEAB | 100 | Zinc triflate, 20 mg | Not IRMOF-3 |

* The word ‘impurity’ refers to the other phase observed by PXRD alongside IRMOF-3 (vide infra).

**Geneal attempted procedure for UiO-66-NH_2_ synthesis.** H_2_ABDC (18.0 mg, 0.099 mmol) and ZrCl_4_ (17.0 mg, 0.073 mmol) were added to THF (2 mL) in a 4 mL vial and sonicated for ~10 minutes (the metal salt did not dissolve with only the linker dissolving). DEAB (12.0 mg, 0.087 mmol) was added to the solution. A cap fitted with an additional Teflon insert with a thickness of 1.87 mm was screwed on then heated to various temperatures and time to form no crystalline product.

**Table S4**: tested synthesis conditions for UiO-66-NH_2_.

| Solvent | Time (days) | Temperature (°C) | Phase |
| --- | --- | --- | --- |
| THF | 1 | 85 | amorphous |
| THF | 2 | 85 | amorphous |
| THF, 10% water | 1 | 85 | amorphous |
| THF, 20% water | 1 | 85 | amorphous |
| THF, 30% water | 1 | 85 | amorphous |
| THF, 40% water | 1 | 85 | amorphous |
| THF, 50% water | 1 | 85 | amorphous |
| THF, 50% water | 7 | 120 | amorphous |

**Table S5**: tested synthesis conditions for Zn-MOF-74 using 10 mg of H_4_DOBDC and 40 mg of Zn(NO_3_)_2_·6H_2_O.

| Solvent | Time (days) | Base Amount | Temperature (°C) | Phase |
| --- | --- | --- | --- | --- |
| THF | 3 | 6 mg DEAB | 100 | Not Zn-MOF-74 |
| THF, water 10% | 3 | 6 mg DEAB | 100 | Not Zn-MOF-74 |
| THF, water 20% | 3 | 6 mg DEAB | 100 | Not Zn-MOF-74 |
| THF, water 30% | 3 | 6 mg DEAB | 100 | Zn-MOF-74 and α phase |
| THF, water 40% | 3 | 6 mg DEAB | 100 | α phase |
| THF, water 50% | 3 | 6 mg DEAB | 100 | α phase |
| THF, water 60% | 3 | 6 mg DEAB | 100 | α phase |
| THF, water 30% | 1 | 6 mg DEAB | 100 | Not Zn-MOF-74 |
| THF, water 30% | 2 | 6 mg DEAB | 100 | Not Zn-MOF-74 |
| THF, water 30% | 3 | 6 mg DEAB | 85 | α phase |
| THF, water 30% | 3 | 24 mg DEAB | 85 | α phase |
| THF, water 30% | 3 | 12 mg DEAB | 100 | Zn-MOF-74 and α phase |
| THF, water 30% | 3 | 18 mg DEAB | 100 | Zn-MOF-74 and α phase |
| THF, water 30% | 3 | 24 mg DEAB | 100 | Zn-MOF-74 and α phase |
| THF, water 30% | 3 | 48 mg DEAB | 100 | Not Zn-MOF-74 |
| THF, water 30% | 3 | 96 mg DEAB | 100 | Not Zn-MOF-74 |
| THF, water 30% | 3 | 30 mg DEAB | 100 | Zn-MOF-74 and α phase |
| THF, water 30% | 3 | 36 mg DEAB | 100 | α phase |
| THF, water 30% | 3 | 42 mg DEAB | 100 | α phase |
| THF, water 30% | 1 | 30 mg DEAB | 120 | β phase |
| THF, water 30% | 3 | 15 μL DBU, 12 mg DEAB | 100 | α phase |
| THF, water 30% | 3 | 7.5 μL DBU, 12 mg DEAB | 100 | α phase |
| THF, water 30% | 1 | 30 μL DBU | 100 | β phase |
| THF, water 30% | 1 | 30 μL DBU | 70 | Zn-MOF-74 and impurities |
| THF, water 30% | 1 | 22.5 μL DBU | 70 | Zn-MOF-74 and impurities |
| THF, water 30% | 1 | 15 μL DBU | 70 | Not Zn-MOF-74 |
| THF, water 30% | 1 | 7.5 μL DBU | 70 | Not Zn-MOF-74 |
| THF, water 30% | 1 | 22.5 μL DBU | 40 | Zn-MOF-74 |
| THF, water 30% | 1 | 22.5 μL DBU | 50 | Zn-MOF-74 and impurities |
| THF, water 30% | 1 | 22.5 μL DBU | 60 | Zn-MOF-74 and impurities |
| THF, water 30% | 1 | 22.5 μL DBU | 80 | β phase |

**Table S6**: tested synthesis conditions for HKUST-1 using 40 mg of H_3_BTC and 80 mg of Cu(NO_3_)_2_·2.5H_2_O.

| Solvent | Time (days) | Base Amount (mg) | Temperature (°C) | Phase |
| --- | --- | --- | --- | --- |
| THF | 1 | 22 mg DEAB | 85 | Not HKUST-1 |
| THF, water 5% | 1 | 22 mg DEAB | 85 | HKUST-1 |
| THF, water 10% | 1 | 22 mg DEAB | 85 | HKUST-1 |
| THF, water 20% | 1 | 22 mg DEAB | 85 | HKUST-1 |
| THF, water 30% | 1 | 22 mg DEAB | 85 | HKUST-1 |
| THF, water 40% | 1 | 22 mg DEAB | 85 | HKUST-1 |
| THF, water 50% | 1 | 22 mg DEAB | 85 | HKUST-1 |
| THF, water 60% | 1 | 22 mg DEAB | 85 | Not HKUST-1 |
| THF, water 20%, HCl two drops | 1 | 22 mg DEAB | 85 | Not HKUST-1 |
| THF, water 20%, HCl two drops | 1 | 22 mg DEAB | 85 | Not HKUST-1 |
| THF, water 30%, HCl two drops | 1 | 22 mg DEAB | 85 | Not HKUST-1 |
| THF, water 30%, HCl two drops | 2 | 22 mg DEAB | 85 | Not HKUST-1 |
| THF, water 40%, HCl two drops | 1 | 22 mg DEAB | 85 | Not HKUST-1 |
| THF, water 50%, HCl two drops | 1 | 22 mg DEAB | 85 | Not HKUST-1 |
| THF, water 50%, HCl two drops | 1 | 22 mg DEAB | 100 | HKUST-1 |

**Table S7:** tested synthesis conditions for MOF-177 using 10.0 mg of H_3_BTB

| Solvent | Time (days) | base amount (mg) | Metal Salt | Temperature (°C) | Amount of Metal (mg) | phase |
| --- | --- | --- | --- | --- | --- | --- |
| THF | 1 | 3 | Zinc nitrate | 85 | 40 | Not MOF-177 |
| THF | 2 | 3 | Zinc nitrate | 85 | 40 | Not MOF-177 |
| THF | 1 | 3 | Zinc triflate | 85 | 40 | Not MOF-177 |
| THF | 2 | 3 | Zinc triflate | 85 | 40 | Not MOF-177 |
| THF | 4 | 3 | Zinc nitrate | 60 | 40 | Not MOF-177 |
| THF | 4 | 3 | Zinc triflate | 60 | 40 | Not MOF-177 |
| THF | 2 | 6 | Zinc nitrate | 60 | 40 | Not MOF-177 |
| THF | 2 | 6 | Zinc triflate | 60 | 40 | Not MOF-177 |
| THF | 4 | 3 | Zinc nitrate | 85 | 49 | Not MOF-177 |
| THF | 4 | 3 | Zinc nitrate | 85 | 40 | Not MOF-177 |
| THF | 4 | 3 | Zinc nitrate | 85 | 37 | Not MOF-177 |
| THF | 2 | 3 | Zinc nitrate | 120 | 40 | Not MOF-177 |

**3. Powder X-ray Patterns (PXRD)**

**Figure S1:** PXRD Patterns for attempted MOF-5 synthesis in 100 % acetonitrile (navy), 100 % ethanol (blue), 100 % ethyl acetate (light red), and 100 % THF (green) compared to the simulated powder of MOF-5 (dark red).

**Figure S2:** PXRD Patterns for attempted MOF-5 synthesis in 100 % THF (dark red), 100 % ethanol (green), 100 % ethyl acetate (blue), and 100 % acetonitrile (light red) with the addition of 1 equiv. diethylamine relative to H_2_BDC compared to the simulated powder of MOF-5 (navy).

**Figure S3:** PXRD Patterns for attempted MOF-5 synthesis in 10% ethanol, 90% DEF (blue), 50% ethanol, 50% DEF (red) and 60% ethanol, 40% DEF (green) compared to the simulated powder of MOF-5 (navy).

**Figure S4:** PXRD Patterns for attempted MOF-5 synthesis in 10% propylene glycol, 90% DEF (navy), 50% propylene glycol, 50% DEF (red) with baseline corrected compared to the simulated powder of MOF-5 (blue) and MOF-69c (green).

**Figure S5:** The experimental PXRD pattern for attempted MOF-5 synthesis in 10% acetonitrile, 90% DEF (blue) compared to the simulated powder of MOF-5 (navy) and MOF-69c (green). The experimental PXRD pattern for 50% acetonitrile, 50% DEF (pink) compared to PNMOF-1 simulated with (0 2 0) preferential orientation. The experimental powder pattern for attempted MOF-5 synthesis in 70% acetonitrile, 30% DEF (yellow).

**Figure S6:** PXRD Patterns for attempted MOF-5 synthesis in 50% ethyl acetate, 50% DEF (navy) and 70% ethyl acetate, 30% DEF (red) compared to the simulated powder of MOF-5 (blue) and MOF-69c (green).

**Figure S7:** PXRD Patterns for attempted MOF-5 synthesis in 10% DMSO, 90% DEF (navy) and 50% DMSO, 50% DEF (red) compared to the simulated powder of MOF-5 (blue) and MOF-69c (green).

**Figure S8:** PXRD Pattern for IRMOF-3 synthesis in 100% THF after washing 3× with THF (blue) compared to simulated IRMOF-3 pattern (green).

**4. Crystal Pictures**

**Figure S9:** light microscopy of IRMOF-5 crystals synthesized in 100% THF using DEAB (left) and diethylamine (right) at 20× objective.

**Figure S10:** light microscopy of attempted MOF-5 synthesis at 10% DMSO, 90% DEF under 5× objective.

**Figure S11:** light microscopy of attempted MOF-5 synthesis at 10% ethanol, 90% DEF under 5× objective.

**Figure S12:** light microscopy of attempted MOF-5 synthesis at 50% propylene glycol, 50% DEF under 5× objective.

**Figure S13:** light microscopy of IRMOF-3 synthesis in 100% THF and 6.2 mg of DEAB after washing 3× with THF and dying with a saturated solution of Nile red. The clear cubic crystals (IRMOF-3) become blue while the other phase remains light brown suggesting it dense or has small pores.

**5. Characterization Data of DEAB**

**Table 8: crystal structure data of diethylammonium bicarbonate**

|  | Diethylammonium bicarbonate |
| --- | --- |
| Empirical formula | C_5_H_13_NO_3_ |
| Formula weight/ g/mol | 135.16 |
| Temperature/K | 293(2) |
| Crystal system | monoclinic |
| Space group | *P*2_1_/n |
| a/Å | 7.3585(4) |
| b/Å | 8.3829(3) |
| c/Å | 13.5760(5) |
| α/° | 90 |
| β/° | 95.435(4) |
| γ/° | 90 |
| Volume/Å^3^ | 833.68(6) |
| Z | 4 |
| ρcalc g/cm^3^ | 1.077 |
| μ/mm^‑1^ | 0.740 |
| Reflections collected | 5432 |
| Independent reflections | 1522 |
| Data/restraints/parameters | 1522/0/86 |
| Goodness-of-fit on F2 | 1.121 |
| Final R indexes [I>=2σ (I)]  R1/wR2 | 0.0588/0.2008 |
| Final R indexes [all data]  R1/wR2 | 0.0661/0.2136 |
| Rint/Rsigma | 0.0201/ 0.0216 |

**Figure 14**. ORTEP diagram for diethylammonium bicarbonate collected at 293(2) K with thermal ellipsoids at 50% probability.

**Figure S15:** ^1^H NMR spectrum of DEAB using D_2_O as the solvent

**Figure S16:** ^13^C NMR spectrum of DEAB using D_2_O as the solvent

**Figure S17:** IR spectrum of DEAB

**Figure S18:** PXRD of DEAB experimental (green) compared to simulated (blue).

6. **N_2_ Sorption Isotherms**

Surface area= 1250.314 m^2^/g

R= 1.0000

C constant= 24961.923

**Figure S19:** Nitrogen adsorption-desorption isotherm at 77K for activated HKUST-1 synthesized in THF.

Surface area= 270.331 m^2^/g

R= 0.99998

C constant= 861.529

**Figure S20:** Nitrogen adsorption-desorption isotherm at 77K for activated IRMOF-3 synthesized in THF.
